# Prospects for Using Computer Accommodography to Predict Myopia Development in Young Adults

**DOI:** 10.3390/life14030324

**Published:** 2024-02-29

**Authors:** Roman Zelentsov, Liliya Poskotinova, Alexandra Moiseeva, Alexander V. Kudryavtsev

**Affiliations:** 1 Department of Family Medicine and Internal Diseases, Northern State Medical University, 163069 Arkhangelsk, Russia; zelentsovrn@gmail.com (R.Z.); ozhiginas73@gmail.com (A.M.); 2 Biorhythmology Laboratory of the Institute of Environmental Physiology, N. Laverov Federal Center for Integrated Arctic Research of the Ural Branch of the Russian Academy of Sciences, 163001 Arkhangelsk, Russia; liliya200572@mail.ru; 3 Department of Community Medicine, UiT The Arctic University of Norway, N-9037 Tromsø, Norway

**Keywords:** myopia, computer accommodation, computer visual syndrome

## Abstract

The diagnostic value of the computer accommodation method remains insufficiently studied. Accommodative and refractive error is a common problem, accounting for 23% of the world’s population. The aim of the study was to investigate the objective parameters of accommodative insufficiency in young people with and without myopia. A cross-sectional study was carried out using a random sample of 116 of university students at the age of 21–23 years. Normal ranges for accommodation parameters in non-myopic participants were defined by 10th and 90th percentile values. The normal ranges were from −0.17 to −0.38 conventional units (c.u.) for accommodative response coefficient (ARC), from 0.08 to 0.41 c.u. for deviation of ARC (σARC), from 0.0 to 0.43 c.u. for accommodogram growth coefficient (AGC), from 54.26 to 58.55 microfluctuations per minute (mcf/min) for coefficient of microfluctuations (CMF), and from 2.58 to 5.26 c.u. for deviation of CMF (σCMF). Signs of computer visual syndrome were observed in 40.9% of non-myopic participants, eye strain in 11.9%, accommodation cramp in 4.5%, and absence or little accommodative response in 3.6%. Therefore, computer accommodation assessment allowed the detection of young people with an increased risk of myopia among those without this ophthalmic pathology.

## 1. Introduction

The diagnostic value of the computer accommodography method remains insufficiently assessed and acknowledged. At the same time, accommodation is an important physiological mechanism for ensuring optimal quality of vision at different distances. Disorders of accommodation and refraction in young people are highly prevalent [1,2]. This is due to the high daily load on the human eyes, including the active use of the internet and digitalization of the educational process [3,4].

Accommodative and refractive error is a common problem, accounting for 23% of the world’s population [5]. Myopia is one of the most common causes of vision loss, while uncorrected myopia is the major cause of decreased distance visual acuity. The observed increase in global myopia prevalence since 2000 and a forecast for the period until 2050 indicate the ongoing prevalence growth [5]. In East Asia, the prevalence of myopia among young people is 80–90%, and myopia is the leading cause of blindness in the region [6]. In the Russian Federation, myopia ranks second in terms of childhood disability and third in terms of disability among the entire population [7]. High myopia accounts for 12% of blindness and low vision cases, for 26.4% of total disability cases in children and for 19% in adults [8].

A study conducted in the Arkhangelsk region of Russia showed that approximately half of all cases of myopia are detected in childhood [9]. The largest proportion of new cases of low myopia was found at the age of 7–14 years, in both males and females. A marked increase in the prevalence of new cases of moderate and high myopia was observed at the age of 10–14 years, whereas at the age of 15–17 years half of the newly diagnosed cases had high myopia. The largest proportion of newly diagnosed myopia cases in adults was observed at the age of 18–29 years, both in women (35%) and men (60%). The study in the Arkhangelsk region also demonstrated a shift towards an earlier manifestation of myopia. Overall, it indicated a high prevalence of myopia in the region and outlined the need for improved early detection through assessing the predictors.

The etiology of myopia is complex and includes environmental factors such as intensive visual activity, which plays an important role [1,2,10]. Studies by E.S. Avetisov in the 1980s have shown that accommodation acts as a regulator of refractogenesis and is one of the key etiological components in myopia development [11,12]. According to the author, eye growth is not a simple increase in its size, but is the formation of the eyeball as a complex optical system under the influence of hereditary and environmental factors. For individuals with a weak accommodative ability, intensive near-visual work (with proximity of the eyes to an object requiring accommodation) becomes an unbearable load for the eyes. In such circumstances, the ciliary muscle continuously signalizes the eye growth control center, prompting it to change the optical system so as to adapt to work at close distance without accommodation stress. The latter is naturally achievable through a moderate lengthening of the anteroposterior axis of the eye. Therefore, myopia manifests as an adaptive reaction, which develops through the lengthening of the eyeball in response to the accommodation stress. Weakness in the supporting connective tissue and other factors may contribute the development myopic refraction in those with weak accommodative ability. Complementary to E.S. Avetisov, the later studies of E. Ong and K.J. Ciuffreda [13] have shown that near-visual work causes unstable myopia by forming a short-term myopic point, which further progresses to myopia with continued intensive visual work at a close distance. A study by K.J. Ciuffreda et al. [14] has also demonstrated that an unstable myopia can be caused by prolonged focus on an approaching object because of the adaptive reaction of the accommodative response.

Individuals whose professional daily activities imply a substantial visual workload are defined as having visually intense work. Professional users of personal computers (PCs) have particularly high risk for the development and progression of myopia, which is commonly preceded by computer visual syndrome (CVS) [15,16]. This syndrome is defined as having objectively measurable or subjectively perceived local ocular symptoms (tension, pain, dryness, irritation, and burning), visual symptoms (blurred vision and double vision), and musculoskeletal symptoms (neck, shoulder, and back pain) [17]. CVS is a growing public health concern as an increase in the prevalence of this condition not only leads to a growth in the prevalence of ophthalmic pathology, but is also a risk factor for a significant decrease in labor productivity [18,19].

Despite the importance of considering accommodation parameters at a vision check, their quantitative assessment has not yet become part of common clinical practice, and there are no standardized approaches. Normative values for selected accommodation parameters, such as accommodative response coefficient indicators, accommodogram growth coefficient, and coefficient of microfluctuations, have been described for children [20]. However, there are no available data on the diagnostic significance of computer accommodography for the adult population.

The computer accommodography method allows the measuring of the earlier mentioned accommodative response coefficient (ARC), coefficient of microfluctuations (CMF), and accommodogram growth coefficient (AGC). ARC is an indicator of the degree of tension of the ciliary muscle in response to a stimulus. CMF is an indicator of the severity of the high-frequency component of ciliary muscle contractions. AGC characterizes the accommodation growth, gradualness of the accommodation tension, and its stability.

In this study, we assessed accommodation parameters in a random sample of young adults at the age of 21–23 years, comprising participants with different degrees of myopia and those without ophthalmic pathology. The aim was to describe the normal ranges of accommodation parameters in young people without myopic pathology and to investigate the performance of the computer accommodography method in the identification of young people with increased myopia risks.

## 2. Materials and Methods

The study had a cross-sectional design. A sample of young people aged 21–23 years was drawn from students of the Federal State Budget Educational Institution of Higher Education “Northern State Medical University” of the Ministry of Healthcare of the Russian Federation, Arkhangelsk, Russia (hereafter NSMU). Participants were recruited from students undergoing a routine ophthalmic examination at the consultative and diagnostic polyclinic of NSMU between 28 September and 28 October 2023. The ophthalmologic status of participants was unknown to researchers at the time of invitation, so myopes and non-myopes had equal chance to be invited. The exclusion criteria were a self-reported mental disorder (ICD-10 diagnoses: F00-F99—mental and behavioral disorders), a neurological disease (a medical record of acute cerebrovascular accident or traumatic brain injury), a diagnosed eye disease (ICD-10 diagnoses: H00-H99—diseases of the eye and adnexa) except for H52—disorders of refraction and accommodation, and self-reported symptoms of acute infections or pain syndrome of any etiology the day before or immediately before the examination.

By default, all participants were tested for uncorrected visual acuity (UCVA) and best corrected visual acuity (BCVA) using the Sivtsev–Golovin table and clinical refraction was measured in diopters. Positive relative accommodation (PRA) was the only measure of the accommodative function in the study, which is conventionally used at a routine eye exam in Russia.

For a more comprehensive assessment of the accommodative function within the study, the participants underwent computer accommodography using the Acomoref-2 (Righton, Japan). The results of computer accommodography were received as graphically displayed accommodograms, which included color palettes with the severity of high-frequency microfluctuations reflected from green (normal) to red (pronounced tension of the ciliary muscle). These accommodograms also illustrated the nature of the accommodative response (AR, color columns) in accordance with the presented accommodative stimulus (AS, contour columns).

According to the manufacturer’s instructions [21], the graphically displayed accommodograms were divided into the following groups: 1—normal accommodative response; 2—absence of accommodative response; 3—CVS; 4—eye strain; and 5—accommodation cramp. A normal accommodogram was characterized by an increasing, stable course of the curve, AR value of less than the accommodative stimulus, and the palette of microfluctuations represented in green and yellow-green with possible single splashes of red at the last steps of the maximum voltage of accommodation. An accommodogram for CVS had an increasing, stable form of a curve, and the palette of microfluctuations was presented in yellow-orange and red, with isolated possible splashes of green. An accommodogram for a habitually excessive tension and spasm of accommodation was characterized by an unstable increasing course of the curve, and the palette was represented in red-orange. An accommodogram in the absence of an accommodative response was characterized by a significantly lower AR than the accommodative stimulus, a “flat” course of the curve in the form of a plateau, and the color palette was represented in green.

Along with the described qualitative assessments of computer accommodograms, computer accommodography with Acomoref-2 included automatic calculation of the following quantitative accommodation indicators: accommodative response coefficient (ARC), deviation of accommodative response coefficient (σARC), accommodogram growth coefficient (AGC), coefficient of microfluctuations (CMF), and deviation of CMF (σCMF) [22]. De facto, the ARC was calculated as (AR − R)/(AS − R), where AR is the accommodative response in diopters, AS was the accommodative stimulus in diopters, and R was the eye’s own refraction. Based on the data of each accommodogram, the average ARC was calculated using the formula ARCavg = ∑ARC/n, where ARCavg is the average value of the ARC of the accommodogram, ∑ARCn is the sum of ARC of all measurement columns, and n is the number of columns. To assess the stability of the accommodogram, we applied the formula σARC = √(∑(ARCi − ARCavg)2/n). To assess the growth/decrease of the accommodogram, we used the growth coefficient (GC) of the accommodogram, which was calculated as GC = n∆AR/n, where n∆AR is the number of values of non-negative values of ∆AR, i.e., AR_i_ − AR_i−1_ ≥ 0, and n is the total number of measurements during the examination. The gradualness of the accommodation tension was estimated by the ratio of the two parameters presented above. To assess the high-frequency component of the accommodation fluctuations, the coefficient of microfluctuations of the accommodation fluctuations was calculated as CMF = HFCamp = ∑HFCn/n, as well as σHFC, where HFCn is the high-frequency component of the accommodation fluctuations of each measurement [22].

Following the examination, the study participants were divided into 2 groups—those with myopia and those without. Subsequently, the degrees of myopia were assessed using autorefractometry data and categorized as follows: low—from −0.5 to −3.0 diopters; moderate—from −3.25 to −6.0 diopters; and high—below −6.25 diopters [23,24]. The normal values of positive relative accommodation for the age of 21–23 years were defined as 3.0–5.0 diopters (Ye.S. Avetistov and K.M. Matz, 1971: O.V. Proskurina, S.Yu. Golubeva et al., 2012) [25]. CMF, σCMF, AGC, ARC, and σARC were analyzed as continuous variables.

Categorical variables were presented as absolute values with percentages. Comparisons of proportions in groups were made using Pearson’s χ2 test. The normality of continuous variables was assessed using the Shapiro–Wilk test, and they were presented as means with standard deviations (M ± SD) or as medians (Me) and with 25th and 75th percentiles (P25–P75), depending on the distributions. Normal ranges for accommodation indicators were defined by 5th and 95th percentiles (P5–P95). The values were considered low-normal if falling between 5th and 10th percentiles (P5–P10), normal if between 10th and 90th percentiles (P10–P90), and high-normal if between 90th and 95th percentiles (P90–P95) [26]. Comparisons of studied groups on accommodation parameters were performed using the Kruskal–Wallis test. Trends were assessed using Jonckheere–Terpstra test. Differences and trends were considered significant at *p* < 0.05. Statistical analysis was performed using Stata 17.0 (StataCorp, College Station, TX, USA).

The study was approved by the Local Ethics Committee of Federal State Budget Educational Institution of Higher Education “Northern State Medical University” of the Ministry of Healthcare of the Russian Federation (Protocol No. 06/09-23 dated 27 September 2023).

## 3. Results

In total, 116 participants (232 eyes) were examined. The average age was 22.3 ± 0.1 years. In 48.3% of total observations (110 eyes) no ophthalmic pathology was found. The presence of myopic refraction was detected in 51.8% of observations (118 eyes). The refraction in this group was −1.54 ± 0.13 diopters. The distribution of the sample by sex and myopic refraction is presented in Table 1.

Most of the myopia cases were categorized as low and moderate myopia (36.2% and 10.8%, respectively). No ophthalmic pathology was found in 48.7% of total eyes examined.

The unaided visual acuity was significantly different between non-myopic participants and those with different myopia levels (Table 2).

The median of uncorrected visual acuity was 1.00 in the non-myopic group, 0.20 in the group with low myopia (from −0.5 to −3.0 diopters), 0.09 among those with moderate myopia (from −3.25 to −6.0 diopters), and 0.04 among those with high myopia (below −6.25 diopters). Best corrected visual acuity was not measured in the group without myopia. It differed between the three groups with myopia, being the lowest among those with high myopia.

Positive relative accommodation (PRA, the non-computer-based parameter) had a median of −4.42 diopters in the group without ophthalmic pathology. In those with myopia, it was lower. The median was −3.65 diopters in participants with low myopia, and −3.60 diopters and −3.17 diopters in those with moderate and high myopia, respectively.

When analyzing the deviation of accommodative response coefficient (σARC), we found it was the highest in the group without ophthalmic pathology. In the groups with myopia, the parameter had lower values (*p* = 0.002).

The group with moderate myopia had lower values of accommodogram growth coefficient (AGC) compared with the group without ophthalmic pathology. However, the values of this parameter in the group with high myopia had the highest value. The differences between groups were significant (*p* = 0.012).

**Figure 1:**
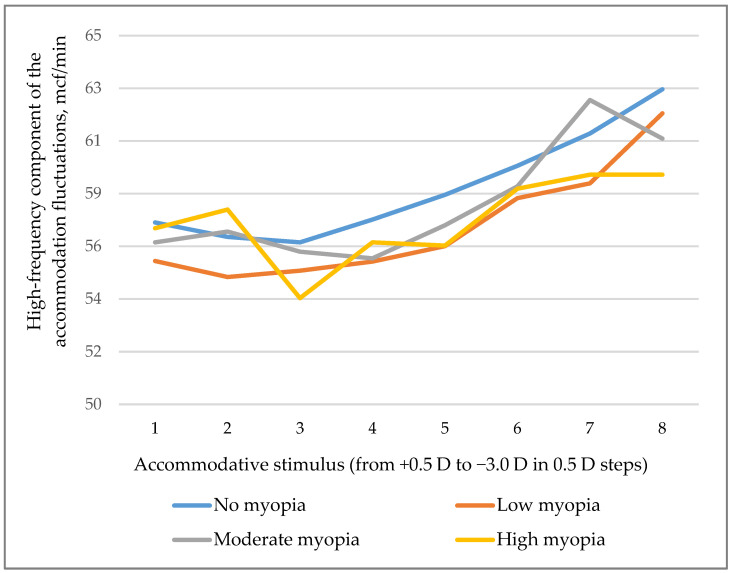
Changes in microfluctuation per minute with an increase in the accommodative stimulus in non-myopic young people aged 21–23 years and in those with myopia.

When assessing the coefficient of microfluctuations (CMF), we found that the frequency of ciliary muscle contractions was reduced in groups with low, medium, and high myopia compared with the group without ophthalmic pathology. The microfluctuation frequency was decreasing along with the increasing myopia degree (p_trend_ = 0.002). The differences between groups are illustrated in Figure 1.

When assessing the values of accommodative response coefficient (ARC) and deviations of CMF, we found no statistically significant differences. However, the median values of ARC tended to increase when comparing the group non-myopic to the groups with increasing myopia degree.

**Figure 2:**
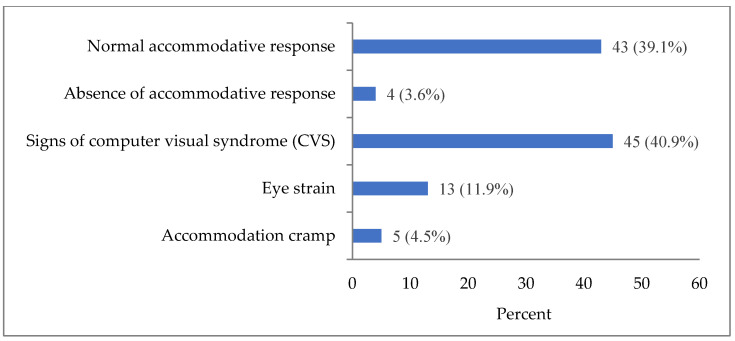
Distribution of the number of accommodation disorders according to the results of computer accommodation in the group of non-myopic participants (number of analyzed accommodograms), Abs. (%).

In the non-myopic group, the following accommodation patterns were identified: normal accommodative response, absence of accommodative response, signs of computer visual syndrome (CVS), eye strain, and accommodation cramp (Figure 2). Normal accommodative response was present only in 39.1% of observations in the group without ophthalmic pathology, including those without refractive errors.

Accommodation pattern with signs of CVS was observed in 40.9% of non-myopic participants, eye strain in 11.9%, accommodation cramp in 4.5%, and absence of (little) accommodative response in 3.6%.

To assess the normal accommodation parameters for the age group of 21–23 years, we selected study participants with normal accommodative response and without refractive errors. For the group of those who met both criteria, the main studied coefficients of accommodograms (ARC, σARC, AGC, CMF, and σCMF) were presented as mean values and as the 5th, 10th, 25th, 50th, 75th, 90th, and 95th percentiles (Table 3). Based on values of the 10th and 90th percentiles, the following ranges were defined as normal for the studied age group: ARC from −0.17 to 0.38 conventional units (c.u.); σARC from 0.08 to 0.41 c.u.; AGC 0.0–0.43 c.u.; CMF from 54.26 to 58.55 mcf/min.; and σCMF from 2.58 to 5.26 c.u.

As can be seen from the graph in Figure 2, the largest proportion of the studied sample (40.9%) showed signs of CVS. For this reason, the main studied coefficients of accommodograms (ARC, σARC, AGC, CMF, and σCMF) were also investigated and presented as mean values and as the 5th, 10th, 25th, 50th, 75th, 90th, and 95th percentiles for the group with CVS signs (Table 4). The 10th–90th percentile ranges of CMF (from 56.83 to 62.49 mcf/min), σCMF (from 2.95 to 5.66 c.u.), and σARC (from 0.11 to 0.66 c.u) values were higher than in the group and with normal accommodative response, while the range of ARC values (from −0.37 to 0.48 c.u.) was wider. There was no difference between the ranges of AGC.

The distributions of computer accommodation coefficients in non-myopic participants with eye strain and accommodation cramp were not analyzed because of the limited numbers of cases.

## 4. Discussion

In this study, we presented empirically defined normal ranges of the main accommodation indicators for non-myopic individuals in the age of 21–23 years. For the accommodative response coefficient (ARC), the normal values ranged from −0.17 to 0.38 c.u.; for deviation of accommodative response coefficient (σARC) from 0.08 to 0.41 c.u.; for accommodogram growth coefficient (AGC), 0.0–0.43 c.u.; for coefficient of microfluctuations (CMF) from 54.26 to 58.55 mcf/min; and for deviation of coefficient of microfluctuations (σCMF) from 2.58 to 5.26 c.u. These normal ranges may be applied in the screening of young adults for early detection of accommodation disorders with a purpose of early myopia prevention.

Accommodation pattern with signs of CVS was observed in 40.9% of non-myopic participants, eye strain in 11.9%, accommodation cramp in 4.5%, and absence or little accommodative response in 3.6%. As is known, these disorders, if left untreated or without correction of visual loads, can lead to the development of myopia [27,28,29,30]. This risk, together with the increasing visual load in the modern life, makes the issue of early detection of these accommodation disorders in young working-age people highly important [31,32]. Since these disorders predispose myopia development and are detectable by accommodography before the deterioration of the visual function, a screening using the accommodography method can be an efficient approach for early detection of accommodation disorders in young adults and timely myopia prevention.

The identified high proportion of participants with signs of CVS among young people with preserved normal visual acuity and the absence of refractive errors may be a consequence of the digitalization of the educational process and the educational environment in a broad sense, which has taken place and is still ongoing [33,34]. This is another indication of the growing relevance of using the computer accommodation method allowing the early identification of changes in the main accommodation parameters in schoolchildren and university students, which, if left unattended, can lead to the development of myopic refraction in early years along with gaining education [27,35].

In this study, we also demonstrated specific features of the accommodogram parameters in individuals with signs of CVS but without myopic refraction. The ranges of CMF, σCMF, and σARC were laying higher, while the range ARC was wider in presence of CVS signs compared with normal values defined for participants with normal accommodative response. These findings in the group with signs of CVS may potentially help to identify individuals with this type of accommodation disorder and at an increased risk of myopia development. For example, non-myopic individuals with CMF, σCMF, or σARC levels exceeding the normal values defined for the group with normal accommodative response can be examined in more depth for CVS and myopia risks. However, longitudinal studies on larger samples must precede the corresponding guidelines.

When assessing the indicators of computer accommodation in participants with myopia, we found a contrary trend towards a decrease in the main indicators of the accommodation diagram: CMF, σCMF, and σARC. Therefore, we can conclude that a decrease in these accommodogram indicators prognostically leads to the progression of myopia in individuals with myopia. These indicators can complement traditionally used methods for assessing accommodative function.

A limitation of the study was the small number of participants with no accommodative response, eye strain, and accommodation cramp. Participants of the study were a continuous sample of medical students 21–23 years old undergoing a routine ophthalmic examination at the university polyclinic in the study period and were considered a random sample. The sampling was random with respect to students of the particular university, as all students undergo ophthalmic examinations periodically, so any student in the age span had a chance to get an ophthalmologist appointment in the study period and be invited to the study. However, the examined medical students may have differed from students of other specialties with respect to the intensity of near-visual work and ophthalmic parameters. Another limitation was a cross-sectional rather than a longitudinal design, which has limited our ability to assess the predictive power of the visual activity and accommodation parameters in myopia development. In addition, we analyzed myopia as a categorical variable (no myopia, low, moderate, and high myopia), although myopia degrees were originally measured in diopters on a continuous scale. Presenting myopia degrees as conventionally used categories was originally selected to make the findings more understandable for practitioners, although using a continuous visual acuity variable could have increased the sensitivity of analyses to detect associations. Given the associations with a rougher categorical scale were present, the scale used was preferred. Finally, the results in this article were presented without considering whether participants were following ergonomic rules in their near-visual work. In the future, we plan to expand the sample for an in-depth longitudinal study of accommodation parameters in a young working-age population. We also plan to further address these parameters in individuals with moderate and high myopia, which may help to better formulate the criteria for early diagnosis and prevention of accommodogram parameters indicative of myopia risks.

Since myopic refraction, as a rule, develops in adolescence, it seems promising to identify the predictors of myopia development using computer accommodation in younger people. The computer accommodography data may also help to identify optimal ergonomic ways to improve visual activity in persons with visually intense work. However, dynamic studies of the accommodography parameters in relation to visual activity and progressing of myopia are needed to strengthen these inferences.

## 5. Conclusions

Our analyses of computer accommodation measurements in myopic and non-myopic individuals have shown a promising performance of the method in identification of young people with increased risk of myopia before the visual function is reduced. For instance, signs of computer accommodation syndrome, a condition known as preceding the development of myopic refraction, was found to be present in 40% of participants without myopia. Therefore, the study demonstrated that computer accommodography could expand the range of ophthalmologist capabilities for early diagnosis and prevention of myopia development in young people and complement traditional methods of studying accommodation. The presented normal ranges of computer accommodation parameters in non-myopic young people with normal accommodative response may be applied in the screening of young adults for early detection of myopia risks and its timely prevention.

## Figures and Tables

**Table 1:** Distribution of refractive errors by severity and sex (numbers of eyes).

Sex	No Myopia	Low Myopia (from −0.5 to −3.0 Diopters)	Moderate Myopia (from −3.25 to −6.0 Diopters)	High Myopia (below −6.25 Diopters)
	Abs (%)			
Male	40 (17.2)	29 (12.5)	9 (3.9)	4 (1.7)
Female	73 (31.5)	55 (23.7)	16 (6.9)	6 (2.6)
Total	113 (48.7)	84 (36.2)	25 (10.8)	10 (4.3)

**Table 2:** Indicators of visual acuity and accommodation in young adults aged 21–23 years.

Indicators, Measuring Units	No Myopia	Low Myopia (from −0.5 to −3.0 Diopters)	Moderate Myopia (from −3.25 to −6.0 Diopters)	High Myopia (below −6.25 Diopters)	*p*
	Me (P25; P75)				
Unaided visual acuity, c.u.	1.00 (1.00; 1.00)	0.20 (0.10; 0.50)	0.09 (0.05; 0.10)	0.04 (0.01; 0.06)	<0.001 **
Best corrected visual acuity, c.u.	–	1.00 (1.00; 1.00)	1.00 (0.90; 1.00)	0.80 (0.70; 1.00)	0.034 *
PRA, diopters	−4.42 (−5.0; −4.0)	−3.65 (−5.0; −2.5)	−3.60 (−5.0; −3.0)	−3.17 (−4.0; −2.0)	<0.001 **
ARC, c.u.	0.10 (−0.09; 0.29)	0.15 (0.0; 0.27)	0.21 (0.02; 0.45)	0.28 (0.11; 0.3)	0.219 **
σARC, c.u.	0.26 (0.15; 0.39)	0.16 (0.11; 0.27)	0.15 (0.10; 0.38)	0.22 (0.09; 0.23)	0.002 *
AGC, c.u.	0.29 (0.14; 0.29)	0.29 (0.16; 0.43)	0.14 (0.14; 0.29)	0.36 (0.14; 0.43)	0.012 *
CMF, mcf/min	58.6 (56.6; 61.4)	56.8 (54.6; 59.5)	56.5 (55.3; 59.6)	57.3 (55.1; 58.4)	0.002 **
σCMF, c.u.	3.84 (3.26; 4.6)	3.74 (2.44; 5.34)	3.66 (2.42; 4.92)	3.25 (1.97; 5.69)	0.759 *

ARC, accommodative response coefficient; σARC, deviation of accommodative response coefficient; AGC, accommodogram growth coefficient; CMF, coefficient of microfluctuations; σCMF, deviation of CMF; PRA, positive relative accommodation; c.u., conventional units; mcf, microfluctuations per minute. Unaided and best corrected visual acuity were assessed using Sivtsev–Golovin table. * Kruskal–Wallis test; ** Jonckheere–Terpstra test.

**Table 3:** Indicators of computer accommodation coefficients in non-myopic participants aged 21–23 years with normal accommodative response.

Indicator	Mean (M)	p5	p10	p25	p50	p75	p90	p95
ARC, c.u.	0.13	−0.20	−0.17	−0.06	0.16	0.29	0.38	0.43
σARC, c.u.	0.22	0.07	0.08	0.12	0.18	0.33	0.41	0.42
AGC, c.u.	0.21	0.00	0.00	0.14	0.29	0.29	0.43	0.43
CMF, mcf/min	56.42	53.64	54.26	54.65	56.55	57.95	58.55	59.68
σCMF, c.u.	3.81	2.13	2.58	3.11	3.80	4.45	5.26	5.53

ARC, accommodative response coefficient; σARC, deviation of accommodative response coefficient; AGC, accommodogram growth coefficient; CMF, coefficient of microfluctuations; σCMF, deviation of CMF; c.u., conventional units; mcf, microfluctuations per minute.

**Table 4:** Indicators of computer accommodation coefficients in non-myopic participants aged 21–23 years with symptoms of computer visual syndrome.

Indicator	Mean (M)	p5	p10	p25	p50	p75	p90	p95
ARC, c.u.	0.07	−0.45	−0.37	−0.14	0.07	0.29	0.48	0.60
σARC, c.u.	0.35	0.10	0.11	0.20	0.31	0.44	0.66	0.71
AGC, c.u.	0.25	0.00	0.00	0.14	0.29	0.29	0.43	0.43
CMF, mcf/min	59.56	55.97	56.83	57.90	59.27	61.59	62.49	63.06
σCMF, c.u.	4.06	2.44	2.95	3.41	3.84	4.73	5.66	5.85

ARC, accommodative response coefficient; σARC, deviation of accommodative response coefficient; AGC, accommodogram growth coefficient; CMF, coefficient of microfluctuations; σCMF, deviation of CMF; c.u., conventional units; mcf, microfluctuations per minute.

## Data Availability

The raw data supporting the conclusions of this article can be made available by the authors on request. All data requests will be guided by protecting of personal information, informed consents of participants, and national legislation.
